# 肝微粒体代谢产物导向的前胡角型吡喃香豆素分离及代谢产物结构鉴定

**DOI:** 10.3724/SP.J.1123.2025.12018

**Published:** 2026-08-08

**Authors:** Yang YANG, Minghao LIU, Hui JIN, Runze SUN, Xingcheng GONG, Wenjing LIU, Yuelin SONG

**Affiliations:** 1. 北京中医药大学，北京 102401; 1. Beijing University of Chinese Medicine，Beijing 102401，China; 2. 河南中医药大学药学院，河南 郑州 450046; 2. Henan University of Chinese Medicine，Zhengzhou 450046，China

**Keywords:** 液相色谱-质谱联用技术, 导向分离, 角型吡喃香豆素, 代谢产物, 结构鉴定, liquid chromatography-mass spectrometry （LC-MS）, guided separation, angular-type pyranocoumarins, metabolites, structural identification

## Abstract

鉴定活性成分代谢产物的结构对于阐明中药体内药效物质至关重要。然而，代谢产物对照品难以获得，仅凭借常规手段难以实现代谢产物结构的准确鉴定。本研究建立了以活性成分肝微粒体代谢产物的保留时间和质谱信息为导向的分离策略，即通过液相色谱-质谱联用技术（LC-MS）追踪，从原药材中定向分离并鉴定目标化合物，将其作为对照品实现肝微粒体代谢产物结构的准确鉴定，进而解析代谢路径。前期研究表明前胡的主要活性成分角型吡喃香豆素（APs）在体内易发生水解反应，本研究选取一对APs同分异构体化合物（+）-白花前胡甲素（（+）-PA）与（+）-北美芹素（（+）-Pte）为底物，分别与人肝微粒体（HLMs）进行体外孵育，利用LC-MS分别检测到8个（包括3个水解产物和5个氧化产物）和9个（包括5个水解产物和4个氧化产物）代谢产物。基于上述策略，从前胡提取物中定向分离制备得到9个APs，包含4对对映体，分别为（3′*S*，4′*S*）-和（3′*R*，4′*R*）-前胡香豆素C（1a/b）、（3′*S*，4′*S*）-和（3′*R*，4′*R*）-前胡香豆素B（2a/b）、（3′*S*，4′*S*）-和（3′*R*，4′*R*）-3′-当归酰氧基-4′-羟基-3′，4′-二氢邪蒿内酯（3a/b）、（3′*S*，4′*S*）-和（3′*R*，4′*R*）-3′-羟基-4′-当归酰氧基-3′，4′-二氢邪蒿内酯（4a/b）和（3′*S*，4′*S*）-isoepoxypteryxin（5）。其中，化合物1b、2b为已知平面结构的新构型。通过与各化学单体进行比对，成功实现了（+）-PA（4个）和（+）-Pte（5个）主要代谢产物的准确鉴定，更揭示了水解、氧化反应及酰基迁移为APs的主要代谢途径。综上，该策略不仅实现了前胡APs的高效分离及（+）-PA和（+）-Pte系列代谢产物结构的准确鉴定，更阐明了APs的主要代谢途径，为中药代谢产物结构的准确鉴定和对照品的获取提供了有效方法。

## Abstract (en)

Identifying the structures of metabolites is of great importance towards elucidating the therapeutic material basis of traditional Chinese medicines （TCMs）. However， current studies lack sufficient reference standards， hindering the structural identification of metabolites. This study establishes a strategy guided by the retention times and mass spectrometric information of microsomal metabolites. By coupling liquid chromatography-mass spectrometry （LC-MS） to trace and isolate target compounds from the raw materials， these compounds served as reference standards to confirm the structures of microsomal metabolites， thereby elucidating the metabolic pathways. *Peucedani Radix* （PR， Chinese name： Qianhu） consists of the dried roots of *Peucedanum praeruptorum* Dunn and contains abundant angular-type pyranocoumarins （APs）， which serve as primary chemical constituents and effective compound clusters as well. From the structural standpoint， C-3′ and C-4′ sites of APs are frequently substituted by acetoxyl， angeloxyl， isovaleroxyl and some other moieties， leading to the extensive occurrences of *regio*-isomers， which not only possess comparable structures but also similar chemical properties. Previous studies have preliminarily unveiled that APs primarily undergo hydrolysis and acyl migration *in vivo*， and these hydrolytic metabolites can be observed in both *in vitro* metabolism and the raw materials. In this study， a pair of *regio*-isomers from PR， *i.e.*， （+）-（3′*S*，4′*S*）-praeruptorin A （（+）-PA） and （+）-（3′*R*，4′*R*）-pteryxin （（+）-Pte）， was incubated separately with human liver microsomes （HLMs） in the presence of necessary cofactors to simulate the metabolic pathways occurring *in vivo*. After carefully processing LC-MS datasets and applying the well-defined mass fragmentation rules of APs， eight metabolites were detected and putatively identified for （+）-PA， including three hydrolysis products together with five oxidation products， whereas nine metabolites were observed and putatively identified for （+）-Pte， encompassing five hydrolysis products and four oxidation products. Noteworthily， phase I metabolism of （+）-Pte in HLMs was profiled for the first time. Various column chromatographic tools， such as silica gel column chromatography， C_18_ reversed-phase chromatography （ODS）， Sephadex LH-20 and semi-preparative high performance liquid chromatography equipped with achiral or chiral columns， were employed to isolate compounds-of-interest from PR extracts under the guidance of LC-MS through taking both retention time and MS/MS information into account. A total of nine AP compounds were successfully purified from PR extracts， and notably， four pairs of AP enantiomers were involved. By matching ^1^H- and ^13^C-NMR spectral information with the data archived in the literature， these compounds were identified as （3′*S*，4′*S*）-qianhucoumarin C （1a）， （3′*R*，4′*R*）-qianhucoumarin C （1b）， （3′*S*，4′*S*）-qianhucoumarin B （2a）， （3′*R*，4′*R*）-qianhucoumarin B （2b）， （3′*S*，4′*S*）-3′-angeloylkhellactone （3a）， （3′*R*，4′*R*）-3′-angeloylkhellactone （3b）， （3′*S*，4′*S*）-4′-angeloylkhellactone （4a）， （3′*R*，4′*R*）-4′-angeloylkhellactone （4b） and （3′*S*，4′*S*）-isoepoxypteryxin （5）. Thereof， compounds 1b and 2b represent new configurational structures of known planar structures. The study summarized mass fragmentation rules for APs and achieved accurate identification of two pairs of *regio*-isomers： qianhucoumarin B and C， and 3′- and 4′-angeloylkhellactone. Thereafter， *in vitro* metabolites of （+）-PA and （+）-Ptewere successfully identified by comparing the retention times and MS/MS information with authentic compounds， including *cis*-khellactone， qianhucoumarin B， qianhucoumarin C， 3′-angeloylkhellactone， 4′-angeloylkhellactone and （3′*S*，4′*S*）-isoepoxypteryxin. Regarding the three hydrolytic metabolites， *i.e.*， *cis*-khellactone， qianhucoumarin B and qianhucoumarin C， they were shared by （+）-PAand （+）-Pte. Hydrolysis and oxidation reactions， as well as acyl migration， served as the primary metabolic pathways for APs in HLMs. In this study， （+）-PAand （+）-Ptewere selected as representatives to demonstrate that it is feasible to obtain their metabolites from PR extracts through LC-MS-guided isolation and purification. After structural identification through analyzing ^1^H- and ^13^C-NMR spectral information， these pure compounds were eligible authentic compounds enabling confirmative structural identification of metabolites， providing pronounced insights into the primary metabolic pathways of APs and offering an effective approach for the accurate identification of metabolite structures through isolating authentic compounds that are available in the original TCM. However， due to differences in metabolism between *in vitro* and *in vivo*， the study failed to construct a definitive structure-activity relationship for the metabolites. Future studies should be devoted to validating these findings through performing *in vivo* investigations.

中药活性成分及其代谢产物是发挥药效的重要组成。研究表明活性成分（如左旋延胡索乙素、松果菊苷、淫羊藿素等）进入体内后，其代谢产物常保留或呈现出更好的生物活性。中药口服给药后，在体内常产生大量的代谢产物，准确鉴定代谢产物结构对于深入阐明中药的药效物质基础至关重要。由于缺乏相关对照品，代谢产物的准确鉴定十分困难。而这些代谢产物可能作为药材原型成分存在于植物中。例如，肉苁蓉主要活性成分松果菊苷在体内可代谢为毛蕊花糖苷，而后者亦为其天然成分之一^［1-3］^。因此，本研究建立了以活性成分肝微粒体代谢产物保留时间和质谱信息为导向的分离策略，通过液相色谱-质谱联用技术（LC-MS）追踪原药材中目标成分，并从原药材中定向分离和鉴定目标化合物，将其作为对照品实现肝微粒体代谢产物的结构鉴定。

前胡（*Peucedani Radix*）为伞形科植物白花前胡（*Peucedanum praeruptorum* Dunn）的干燥根，具有降气化痰、疏散风热等功效，临床多用于痰热咳喘、风热感冒等症^［4］^。角型吡喃香豆素（angular-type pyranocoumarins，APs）作为前胡主要的药效成分，具有祛痰、止咳等活性^［5，6］^。该类化合物常以顺式凯琳内酯（*cis*-khellactone）为母核，吡喃环的C-3′和（或）C-4′常连接乙酰氧基、当归酰氧基等基团，且因手性中心的存在，导致原药材中广泛存在位置异构体和对映异构体，化学组成复杂。课题组前期研究结果表明，前胡主要活性成分白花前胡甲素对映体（（±）-praeruptorin A， （±）-PA）、右旋白花前胡乙素（（+）-praeruptorin B， （+）-PB）等在体内代谢途径主要为酯键水解、氧化反应及酰基迁移^［7，8］^。然而，受限于代谢产物含量极低且缺乏对照品，加之APs成分结构的高度相似性，仅依赖LC-MS技术难以准确区分复杂的同分异构体，导致多数微量代谢产物的结构难以鉴定。结合课题组前期对前胡提取物的定性分析结果^［9，10］^以及文献中已报道的前胡化学成分^［11-13］^，我们推测APs的体内代谢产物同样也作为原型成分存在于前胡原药材中。因此，开发高效的代谢产物对照品获取方法，可以实现APs活性代谢产物的准确鉴定，为前胡药效物质的深入阐明奠定基础。

体外代谢模型，如人肝微粒体（human liver microsomes，HLMs）孵育系统，可以产生多种体内代谢产物，较好地模拟体内代谢过程。因此，为了实现代谢产物的准确鉴定，本研究建立以肝微粒体代谢产物为导向的分离策略：（1）通过HLMs孵育体系获得（+）-PA和（+）-北美芹素（（+）-pteryxin，（+）-Pte）的主要代谢产物，基于LC-MS技术解析其保留时间、特征碎片离子等信息；（2）将这些信息作为导向，在前胡提取物中进行成分匹配；（3）综合运用多种色谱分离技术（如硅胶柱色谱、Sephadex LH-20凝胶柱色谱、反相色谱和手性色谱等）从前胡提取物中定向制备目标APs成分，将其作为对照品实现（+）-PA和（+）-Pte的部分代谢产物的结构鉴定。本研究通过此策略，实现了前胡中APs化合物的高效分离与结构鉴定，进而阐明了（+）-PA和（+）-Pte的代谢路径，为中药药效成分的体内代谢和起效形式研究提供参考。

## 1 实验部分

### 1.1 仪器、试剂与材料

Thermo-Fisher U3000双三元分析型液相色谱仪（美国Thermo-Fisher公司）；SCIEX Triple TOF 6600^+^（美国SCIEX公司）；LC-20AD_XR_型分析型液相色谱仪和LC-20AT型半制备型液相色谱仪（日本岛津公司）；硅胶（200~300 目）和GF_254_硅胶预制板（青岛海洋化工厂）；ODS填料（40~63 μm，德国Merck公司）。

提取分离所用无水乙醇（EtOH）、石油醚（PE）、二氯甲烷（DCM）、甲醇（MeOH）、乙酸乙酯（EtOAc）和正丁醇等试剂均为分析纯（西陇科学股份有限公司）；茴香醛显色剂（天津市福辰化学试剂厂）；质谱级乙腈（ACN）、甲酸、色谱级乙醇、正己烷（*n*-hexane）（美国Thermo-Fisher公司）；水为实验室自制超纯水（18.2 MΩ·cm，美国Millipore公司）。

（+）-白花前胡甲素和顺式凯琳内酯为课题组前期分离获得^［14］^；（+）-北美芹素（纯度≥98%）、Tris-HCl缓冲液（pH 7.4）（上海源叶生物科技有限公司）；氯化镁（MgCl_2_）、还原型辅酶Ⅱ（NADPH）、葡萄糖-6-磷酸（G-6-P）、葡萄糖-6-磷酸脱氢酶（G-6-PD）以及商业化人肝微粒体（美国Sigma公司）。

前胡于2022年11月购自安徽亳州药材市场，由北京大学药学院屠鹏飞教授鉴定为伞形科植物白花前胡的干燥根，标本存放于北京中医药大学北京中医药研究院中药现代研究中心（标本号：2022-SYL-QH1）。

### 1.2 （+）-PA和（+）-Pte在HLMs中的体外孵育

体外孵育体系（终体积为200 μL）由0.05 mol/L Tris-HCl缓冲液（pH 7.4）组成，包括3.3 mmol/L MgCl_2_、1.0 mmol/L NADPH、5.0 mmol/L G-6-P、1.0 U/mL G-6-PD、1 mg/mL HLMs及25 μmol/L底物（（+）-PA和（+）-Pte）。混合液于37 ℃恒温振荡（300 r/min）预孵育5 min后，加入1.0 mmol/L NADPH启动反应。孵育2 h后，用600 μL的EtOAc萃取3次，合并上层溶液，氮气吹干。残渣用50 μL甲醇复溶，经0.22 μm微孔滤膜过滤后，进行LC-MS分析。另设空白对照组，以等体积甲醇代替底物溶液。

### 1.3 LC-MS分析

LC-MS分析采用Thermo Ultimate 3000 UHPLC系统联用SCIEX TripleTOF 6600^+^-MS。色谱柱：Agilent ZORBAX SB-C_18_色谱柱（250 mm×4.6 mm， 5.0 μm）；流动相：0.1%甲酸水溶液（A）和乙腈（B）；梯度洗脱程序：0~35.0 min， 25%B； 35.0~40.0 min， 25%B~45%B； 40.0~42.0 min， 45%B~57%B； 42.0~48.0 min， 57%B~67%B； 48.0~53.0 min， 67%B~95%B； 53.0~53.1 min， 95%B~25%B； 53.1~59.0 min， 25%B； 柱温：35 °C； 流速：1.0 mL/min。Analyst TF 1.7（SCIEX）和Chromeleon（Version 7.2， Thermo-Fisher Scientific）软件分别控制质谱采集和液相程序洗脱。

采用电喷雾离子源（ESI），正离子模式采集。离子源参数设置：喷雾电压为5 500 V；气帘气（CUR）、雾化气（GS1）和辅助加热气（GS2）气压分别设为0.24、0.34和0.34 MPa；离子源温度设为500 ℃。MS^1^全扫描，质量扫描范围为*m*/*z* 100~1 000，MS^2^中设定碰撞能量（CE）为30 eV，质量扫描范围为*m*/*z* 50~1 000。PeakView 1.2（SCIEX）软件用于数据处理。

### 1.4 提取与分离

前胡干燥饮片（5.0 kg），粉碎，用45 L 95%乙醇水溶液加热回流提取3次，每次1 h。合并提取液，减压回收溶剂至无醇味，得总浸膏。将浸膏混悬于5 L蒸馏水中，依次使用5 L石油醚、二氯甲烷、乙酸乙酯和正丁醇萃取，每种溶剂萃取3次。各萃取液减压浓缩后，分别得到石油醚（Fr. P）、二氯甲烷、乙酸乙酯（Fr. E）及正丁醇4个萃取部位。依据1.3节方法，根据肝微粒体代谢产物的保留时间和质谱信息为导向，利用LC-MS追踪前胡各萃取部位中的目标成分，根据分析结果选择石油醚部位和乙酸乙酯部位进行后续分离。

取Fr. E（7.8 g），利用自制硅胶柱（柱子半径（*r*）=2.25 cm，高度（*h*）=20 cm）分步洗脱。首先，以PE-EtOAc系统（20∶1→10∶1→5∶1→1∶1，体积比）进行洗脱，随后替换为DCM-MeOH系统（20∶1→10∶1→5∶1→0∶1，体积比）进行洗脱。洗脱液经TLC检识，将斑点相同的流分合并，编号为Frs. E1~E11。取Fr. E6（57.6 mg）利用Sephadex LH-20凝胶柱色谱纯化，以DCM-MeOH（3∶1，体积比）洗脱，合并相同斑点的流分，编号为Frs. E6a~E6d。取Fr. E6b（18.4 mg）进一步利用半制备液相色谱进行手性分离。Chiral INC正相手性色谱柱Ⅰ（250 mm×4.6 mm，5.0 μm，广州菲罗门科学仪器有限公司）；流动相，EtOH-正己烷（20∶80，体积比）；流速，1.0 mL/min；检测波长，323 nm，分别收集各色谱峰。

取Fr. P（193.8 g）经硅胶柱（*r*=4.5 cm，*h*=100 cm）分离，依次以PE-EtOAc系统（20∶1→10∶1→5∶1→1∶1，体积比）和DCM-MeOH系统（20∶1→10∶1→5∶1→0∶1，体积比）梯度洗脱。TLC检识指导合并，共得到25个流分（Frs. P1~P25）。

取Fr. P16（16.7 g）经硅胶柱（*r*=3.0 cm，*h*=25 cm）分离，以PE-EtOAc系统（10∶1→5∶1→1∶1，体积比）梯度洗脱，TLC检识指导合并，得到13个流分（Frs. P16a~P16m）。

取Fr. P16f（12.0 g）经自制ODS反相柱（*r*=2.5 cm，*h*=15 cm）分离，MeOH-H_2_O系统（1∶1→1∶0，体积比）梯度洗脱，TLC检识指导，得到9个流分（Frs. P16f1~P16f9）。

取Fr. P16f6（92.4 mg）利用Sephadex LH-20色谱柱（瑞典GE Healthcare Bio-Sciences公司）纯化，DCM-MeOH系统（3∶1，体积比）洗脱，TLC检识指导合并，得到2个流分（Frs. P16f6a和P16f6b）。取Fr. P16f6a（52.8 mg）经Phenomenex Luna C_18_ （2）色谱柱（250 mm×10 mm，5.0 μm）制备分离，流动相ACN-H_2_O（70∶30，体积比），流速3.0 mL/min，检测波长323 nm，共收集到4个流分（Frs. P16f6a1~P16f6a4）。

取Fr. P16f6a3（16.0 mg）经Waters SunFire OBD Prep正相制备色谱柱（250 mm × 10 mm，5.0 μm）纯化，流动相EtOH-正己烷（10∶90，体积比），流速3.0 mL/min，检测波长323 nm，分别收集对应色谱峰，获得化合物3和4。化合物3经CHIRALPAK^®^ OX-3正相手性色谱柱Ⅱ（150 mm×4.6 mm，3.0 μm，日本大赛璐公司）拆分，流动相EtOH-正己烷（25∶75，体积比），流速0.5 mL/min，检测波长323 nm，最终收集流分，制得化合物3a和3b。

化合物4经过Chiral INC正相手性色谱柱Ⅰ拆分对映体，流动相EtOH-正己烷（50∶50，体积比），流速0.7 mL/min，检测波长323 nm，最终收集流分，制得化合物4a和4b。

Fr. P16g（0.8 g）经硅胶柱（*r*=1.5 cm，*h*=16 cm）分离，依次以PE-EtOAc系统（20∶1→10∶1→5∶1→1∶1，体积比）和DCM-MeOH系统（10∶1→5∶1→0∶1，体积比）梯度洗脱，TLC检识指导合并，共得到11个流分（Frs. P16g1~P16g11）。

取Fr. P16g6（153.2 mg）经ODS反相柱（*r*=1.0 cm，*h*=13 cm）分离，经MeOH-H_2_O（1∶3→1∶1→1∶0）梯度洗脱，TLC检识指导合并，共得到17个流分（Frs. P16g6a~P16g6q）。其中，Fr. P16g6j（23.8 mg），经Phenomenex Luna C_18_（2）反相制备液相色谱柱纯化，流动相ACN-H_2_O（40∶60，体积比），流速3.0 mL/min，检测波长323 nm，最终制得化合物5。

## 2 结果与讨论

### 2.1 （+）-PA和（+）-Pte的体外孵育产物鉴定

将（+）-PA和（+）-Pte分别与HLMs共孵育，获得二者体外代谢产物，见如图1。结果显示，在（+）-PA和（+）-Pte体系中分别检测到8个（A1~A8）和9个（E1~E9）代谢产物，上述代谢产物的色谱和质谱数据见表1和表2。通过总结APs质谱裂解规律，初步推断代谢产物结构，进一步通过与后续前胡药材中分离获得的标准品进行比对，实现部分代谢产物结构的准确鉴定。

**图1:**
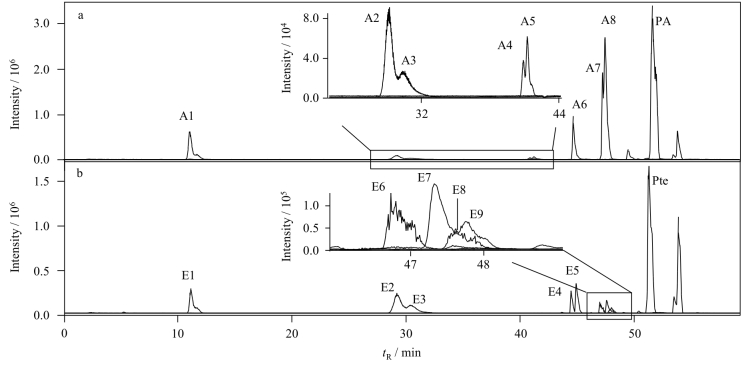
HLMs中（a）（+）-PA和（b）（+）-Pte代谢产物的提取离子流图

**表1:** （+）-PA及其代谢产物（A1~A8）的色谱和质谱数据

Compound	*t*_R_/min	Formula	MS^1^ （*m*/*z*） （quasimolecular ion）	Error/10^-6^	MS^2^ （*m*/*z*）
（+）-PA	51.4	C_21_H_22_O_7_	404.1706 ［M+NH_4_］^+^	0.5	327.1251， 227.0701
			409.1255 ［M+Na］^+^	‒1.0	349.1029， 327.1215， 227.0695， 215.0685
A1	11.0	C_14_H_14_O_5_	263.0921 ［M+H］^+^	2.7	203.0697， 187.0379， 175.0385
			280.1180 ［M+NH_4_］^+^	0.2	263.0912， 203.0696， 175.0386
A2	29.1	C_16_H_16_O_6_	322.1289 ［M+NH_4_］^+^	1.2	245.0804， 203.0695， 187.0376， 175.0385
			327.0842 ［M+Na］^+^	0.9	267.0629， 203.0700
A3	30.3	C_16_H_16_O_6_	322.1281 ［M+NH_4_］^+^	‒1.3	305.1028， 287.0920， 245.0816， 227.0711， 203.0700， 175.0396
			327.0834 ［M+Na］^+^	‒1.6	287.0918， 203.0702
A4	40.7	C_21_H_24_O_9_	438.1761 ［M+NH_4_］^+^	0.6	361.1271， 245.0799， 227.0698
			443.1313 ［M+Na］^+^	0.1	383.1073， 251.0670， 245.0802， 227.0691
A5	40.9	C_21_H_24_O_9_	438.1757 ［M+NH_4_］^+^	‒0.4	361.1269， 317.0998， 245.0803， 227.0701， 175.0384
			443.1313 ［M+Na］^+^	0.1	383.1084， 251.0677， 245.0807， 227.0693
A6	44.5	C_21_H_22_O_8_	420.1651 ［M+NH_4_］^+^	‒0.5	343.1171， 325.1081， 245.0802， 227.0690， 175.0365
			425.1200 ［M+Na］^+^	‒1.6	343.1163， 267.0611， 245.0787， 227.0680， 175.0373
A7	47.0	C_21_H_22_O_8_	420.1659 ［M+NH_4_］^+^	1.4	343.1164， 315.1214， 299.0910， 255.1004， 245.0809， 227.0701
			425.1211 ［M+Na］^+^	1.0	365.0983， 245.0709， 227.0698
A8	47.3	C_21_H_22_O_8_	420.1649 ［M+NH_4_］^+^	‒0.9	343.1168， 255.1009， 245.0804， 227.0699， 175.0382
			425.1202 ［M+Na］^+^	‒1.1	365.0980， 245.0800， 227.0696

**表2:** （+）-Pte及其代谢产物（E1~E9）的色谱和质谱数据

Compound	*t*_R_/min	Formula	MS^1^ （*m*/*z*）（quasimolecular ion）	Error /10^‒6^	MS^2^ （*m*/*z*）
（+）-Pte	51.0	C_21_H_22_O_7_	404.1705 ［M+NH_4_］^+^	0.3	287.0903， 245.0797， 227.0693， 175.0382
			409.1264 ［M+Na］^+^	1.5	309.0729， 245.0805， 227.0693， 175.0381
E1	11.0	C_14_H_14_O_5_	263.0923 ［M+H］^+^	3.4	203.0694， 175.0379
			280.1183 ［M+NH_4_］^+^	1.3	263.0895， 245.0796， 203.0691， 187.0387， 175.0383
E2	29.1	C_16_H_16_O_6_	322.1288 ［M+NH_4_］^+^	0.9	245.0794， 203.0694， 187.0377， 175.0366
			327.0844 ［M+Na］^+^	1.5	267.0611， 203.0688， 187.0436， 175.0380
E3	30.3	C_16_H_16_O_6_	322.1283 ［M+NH_4_］^+^	‒0.7	305.1026， 287.0921， 245.0815， 227.0706， 203.0699， 175.0388
			327.0840 ［M+Na］^+^	0.3	287.0924， 203.0696
E4	44.2	C_21_H_22_O_8_	420.1654 ［M+NH_4_］^+^	0.3	287.0901， 245.0798， 227.0696， 175.0373
			425.1209 ［M+Na］^+^	0.5	309.0724， 245.0798， 227.0689， 175.0378
E5	44.7	C_21_H_22_O_8_	420.1660 ［M+NH_4_］^+^	1.7	287.0902， 245.0803， 227.0695， 175.0379
			425.1202 ［M+Na］^+^	‒1.1	365.0980， 245.0800， 227.0696
E6	46.8	C_19_H_20_O_6_	362.1608 ［M+NH_4_］^+^	2.7	245.0806， 203.0707， 187.0386， 175.0384
			367.1160 ［M+Na］^+^	2.2	267.0624， 203.0693
E7	47.3	C_21_H_22_O_8_	420.1655 ［M+NH_4_］^+^	0.5	287.0903， 245.0801， 227.0691， 175.0380
			425.1208 ［M+Na］^+^	0.3	309.0708， 245.0805， 227.0682
E8	47.6	C_19_H_20_O_6_	362.1606 ［M+NH_4_］^+^	2.2	345.1317， 327.1219， 227.0696， 349.1035， 267.0619
			367.1159 ［M+Na］^+^	‒4.7	
E9	47.8	C_21_H_22_O_8_	420.1647 ［M+NH_4_］^+^	‒1.4	287.0897， 245.0794， 227.0689，
			425.1196 ［M+Na］^+^	‒2.6	367.1176， 309.0723， 245.0768， 227.0703

#### 2.1.1 基于特征碎片离子的位置异构体区分

代谢产物中存在多组易发生互变的位置异构体（如前胡香豆素B/C），难以实现良好的色谱分离，仅凭保留时间无法准确指认。本研究利用APs化合物在ESI下的特征裂解规律进行区分^［9］^：母离子经碰撞诱导解离（CID）后，会在母核吡喃环C-4′处优先发生中性丢失，产生特征碎片离子，再进一步中性丢失C-3′处取代基，产生其他特征碎片离子。因此，首先根据母离子C-4′处中性丢失所产生的特征碎片离子可以确定C-4′处取代基，进一步确定C-3′处取代基，实现位置异构体的区分，具体如下（图2和图3）：（1）当C-4′为羟基时（如前胡香豆素B），［M+NH_4_］^+^离子易丢失NH_3_和H_2_O，生成*m*/*z* 287.092 7的特征碎片离子；（2）当C-4′为乙酰氧基时（如前胡香豆素C），［M+NH_4_］^+^离子易丢失NH_3_和CH_3_COOH，产生*m*/*z* 245.082 2的特征碎片离子。以上规律为后续异构体代谢产物的精准鉴定提供依据。

**图2:**
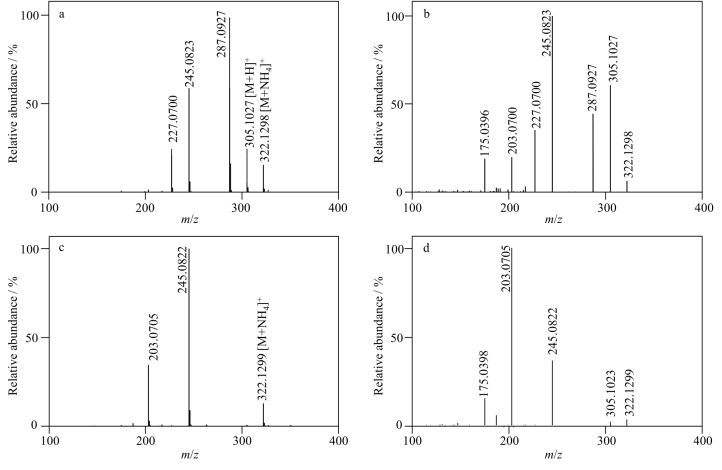
前胡香豆素B的（a）MS^1^和（b）MS^2^质谱图以及前胡香豆素C的（c）MS^1^和（d）MS^2^质谱图

**图3:**
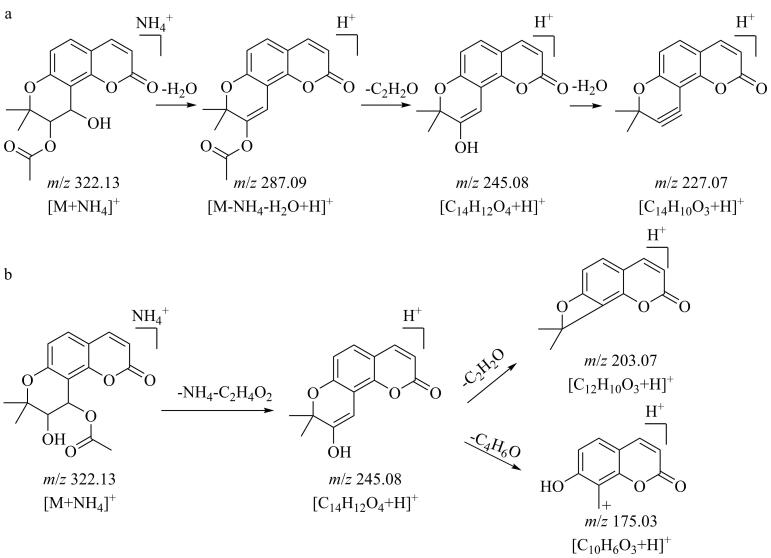
（a）前胡香豆素B和（b）前胡香豆素C可能的裂解途径

#### 2.1.2 （+）-PA的代谢产物鉴定

（+）-PA的8个代谢产物中，包括3个水解产物（A1~A3）和5个氧化产物（A4~A8），见图4和表1。

**图4:**
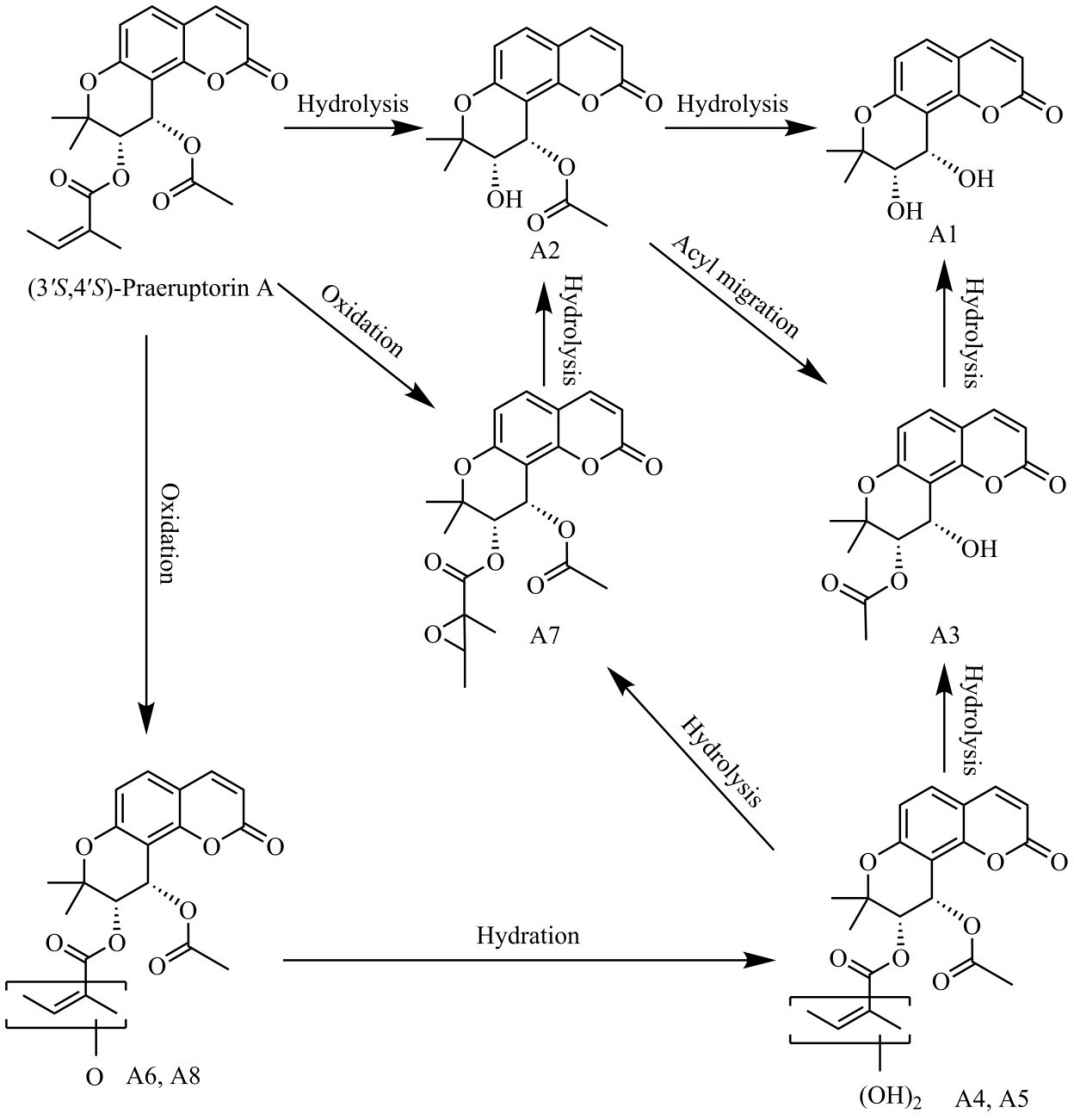
（+）-PA在HLMs中可能的代谢途径

代谢产物A1（*t*_R_=11.0 min）的准分子离子峰为*m*/*z* 263.092 1 ［M+H］^+^。经对照品比对，其保留时间和质谱裂解行为与顺式凯琳内酯完全一致，故将A1准确鉴定为顺式凯琳内酯。

代谢产物A2（*t*_R_=29.1 min）和A3（*t*_R_=30.3 min）为一组同分异构体。基于上述质谱裂解规律，通过检测特征碎片*m*/*z* 245.082 2和287.092 7，结合对照品保留时间，将A2和A3分别鉴定为前胡香豆素C和前胡香豆素B。

代谢产物A4（*t*_R_=40.7 min）和A5（*t*_R_=40.9 min）母离子均为*m*/*z* 443.131 3 ［M+Na］^+^（C_21_H_24_O_9_，误差1.0×10^-7^）。MS^2^中，二者均产生*m*/*z* 383.11的碎片，对应C-4′位中性丢失一分子乙酸，表明C-4′位结构完整；随后产生*m*/*z* 251.07碎片提示氧化反应发生于C-3′侧链。据此推断A4和A5为C-3′侧链双羟基化产物。

代谢产物A6（*t*_R_=44.5 min）、A7（*t*_R_=47.0 min）和A8（*t*_R_=47.3 min）母离子均为*m*/*z* 425.120 0 ［M+Na］^+^（C_21_H_22_O_8_，误差-1.6×10^-6^），推测为单氧化产物。其中，A7与对照品5具有一致的保留时间和质谱裂解行为，故将产物A7鉴定为isoepoxypteryxin，并进一步推测A6和A8为侧链其他位点的氧化异构体。

#### 2.1.3 （+）-Pte的代谢产物鉴定

本研究阐明了（+）-Pte在HLMs中的Ⅰ相代谢过程，共鉴定出9个代谢产物，包括5个水解产物（E1~E3、E6、E8）和4个氧化产物（E4、E5、E7、E9），见图5和表2。

**图5:**
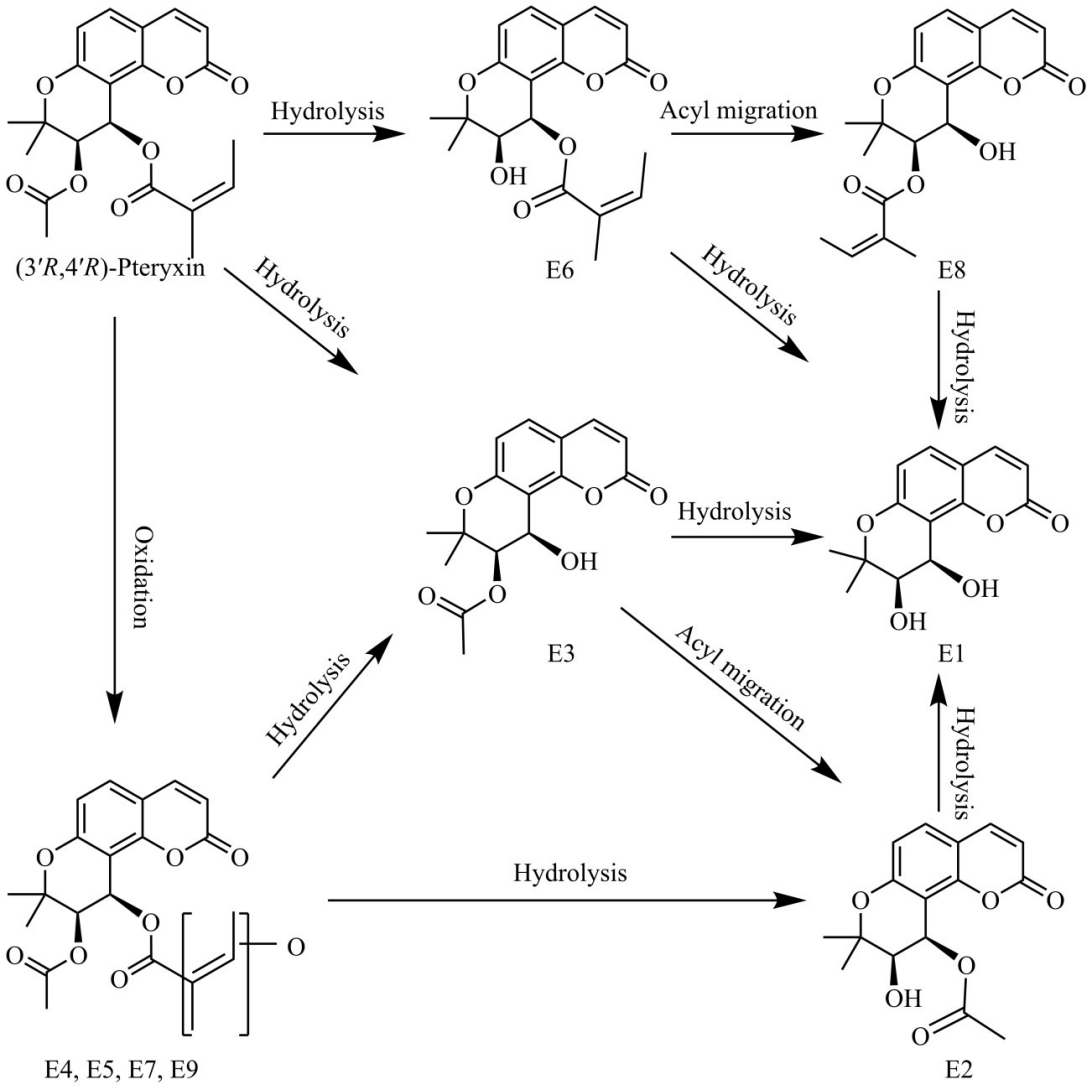
（+）-Pte在HLMs中可能的代谢途径

代谢产物E1、E2和E3分别与（+）-PA的代谢产物A1、A2和A3对应，表明二者存在共同的水解代谢途径。代谢产物E6（*t*_R_=46.8 min）和E8（*t*_R_=47.6 min）为另一组位置异构体。E6和E8分别与对照品4和3具有一致的保留时间和质谱裂解行为，故将E6和E8分别鉴定为3′-羟基-4′-当归酰氧基-3′，4′-二氢邪蒿内酯和3′-当归酰氧基-4′-羟基-3′，4′-二氢邪蒿内酯。此外，氧化产物E4（*t*_R_=44.2 min）、E5（*t*_R_=44.7 min）、E7（*t*_R_=47.3 min）和E9（*t*_R_=47.8 min）的质谱特征提示其氧化位点主要位于C-4′位的当归酰氧基侧链。

### 2.2 前胡提取物中原型成分的分离及结构鉴定

通过对体外代谢产物数据进行分析，表明目标化合物主要富集于石油醚部位（193.8 g）和乙酸乙酯部位（7.8 g），其余二氯甲烷部位（11.0 g）及正丁醇部位（30.3 g）中目标成分含量较低。通过对上述两个部位的系统分离，共得到9个APs（图6）。

**图6:**
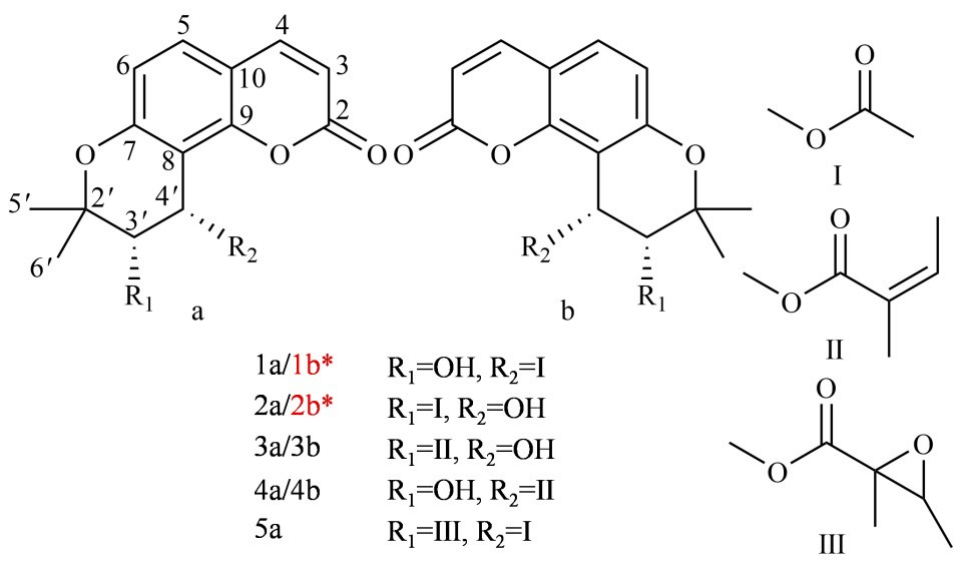
角型吡喃香豆素1~5的化学结构

从乙酸乙酯部位中分离得到4个APs（图7），分别为1a（7.1 mg，*t*_R_=12.2 min）、2a（2.9 mg，*t*_R_=14.0 min）、2b（0.5 mg，*t*_R_=14.6 min）和1b（2.1 mg，*t*_R_=16.1 min）。从石油醚部位分离得到5个APs，分别为3a（1.0 mg，*t*_R_=6.8 min）、3b（0.6 mg，*t*_R_=8.5 min）、4a（4.4 mg，*t*_R_=5.0 min）、4b（2.0 mg，*t*_R_=7.3 min）和5（7.5 mg，*t*_R_=24.6 min）。其中，化合物1b、2b为已知平面结构的新构型。

**图7:**
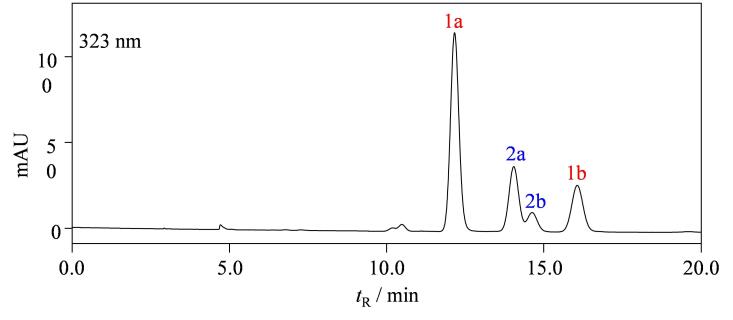
化合物1和2的对映体拆分色谱图

通过^1^H、^13^C-NMR等波谱技术鉴定化合物平面结构，按照文献［^15^］方法，通过Ⅰ相代谢水解反应获得共同产物凯琳内酯，根据凯琳内酯的绝对构型确定化合物的绝对构型，故将化合物1~5分别鉴定为（3′*S*，4′*S*）-和（3′*R*，4′*R*）-前胡香豆素C（1a/1b）、（3′*S*，4′*S*）-和（3′*R*，4′*R*）-前胡香豆素B（2a/2b）、（3′*S*，4′*S*）-和（3′*R*，4′*R*）-3′-当归酰氧基-4′-羟基-3′，4′-二氢邪蒿内酯（3a/3b）、（3′*S*，4′*S*）-和（3′*R*，4′*R*）-3′-羟基-4′-当归酰氧基-3′，4′-二氢邪蒿内酯（4a/4b）和（3′*S*，4′*S*）-isoepoxypteryxin（5）。

化合物1（1a/1b**）** 白色粉末；ESI-MS *m/z* 327.085 4 ［M+Na］^+^，分子式为C_16_H_16_O_6_（理论值为*m*/*z* 327.038 9，误差+4.6×10^‒6^）。^1^H-NMR （CDCl_3_， 400 MHz）： *δ*_H_ 7.61 （1H， d， *J*=9.5 Hz， H-4）， 7.35 （1H， d， *J*=8.6 Hz， H-5）， 6.80 （1H， d， *J*=8.6 Hz， H-6）， 6.43 （1H， d， *J*=4.7 Hz， H-4′）， 6.23 （1H， d， *J*=9.5 Hz， H-3）， 4.00 （1H， d， *J*=4.7 Hz， H-3′）， 2.23 （3H， s， 2‴-CH_3_）， 1.46 （3H， s， 5′-CH_3_）， 1.44 （3H， s， 6′-CH_3_）。^13^C-NMR （CDCl_3_， 100 MHz）： *δ*_C_ 159.9 （C-2）， 113.0 （C-3）， 143.3 （C-4）， 129.2 （C-5）， 114.5 （C-6）， 157.0 （C-7）， 106.9 （C-8）， 154.3 （C-9）， 112.4 （C-10）， 78.8 （C-2′）， 71.2 （C-3′）， 63.0 （C-4′）， 25.2 （C-5′）， 21.6 （C-6′）， 172.0 （C-1‴）， 20.9 （C-2″）。以上数据与文献报道基本一致^［11］^，将化合物1a和1b分别鉴定为（3′*S*，4′*S*）-前胡香豆素C和（3′*R*，4′*R*）-前胡香豆素C。

化合物2（2a/2b） 白色粉末；ESI-MS *m*/*z* 327.085 7 ［M+Na］^+^，分子式为C_16_H_16_O_6_（理论值为*m*/*z* 327.038 9，误差+5.5×10^‒6^）。^1^H-NMR （CDCl_3_， 400 MHz）： *δ*_H_ 7.64 （1H， d， *J*=9.5 Hz， H-4）， 7.34 （1H， d， *J*=8.6 Hz， H-5）， 6.80 （1H， d， *J*=8.6 Hz， H-6）， 6.26 （1H， d， *J*=9.5 Hz， H-3）， 5.42 （1H， d， *J*=4.9 Hz， H-3′）， 5.19 （1H， d， *J*=4.9 Hz， H-4′）， 2.18 （3H， s， 2″-CH_3_）， 1.48 （3H， s， 5′-CH_3_）， 1.40 （3H， s， 6′-CH_3_）。^13^C-NMR （CDCl_3_， 100 MHz）： *δ*_C_ 160.3 （C-2）， 112.7 （C-3）， 143.8 （C-4）， 128.7 （C-5）， 114.7 （C-6）， 155.9 （C-7）， 110.1 （C-8）， 154.4 （C-9）， 112.3 （C-10）， 77.7 （C-2′）， 72.6 （C-3′）， 60.2 （C-4′）， 25.3 （C-5′）， 22.6 （C-6′）， 170.3 （C-1″）， 20.7 （C-2″）。以上数据与文献报道基本一致^［11］^，将化合物2a和2b分别鉴定为（3′*S*，4′*S*）-前胡香豆素B和（3′*R*，4′*R*）-前胡香豆素B。

化合物3（3a/3b） 白色粉末；ESI-MS *m*/*z* 367.117 4 ［M+Na］^+^，分子式C_19_H_20_O_6_（理论值为*m*/*z* 367.115 2，误差+6.0×10^‒6^）。^1^H-NMR （CDCl_3_， 600 MHz）： *δ*_H_ 7.64 （1H， d， *J*=9.5 Hz， H-4）， 7.34 （1H， d， *J*=8.6 Hz， H-5）， 6.80 （1H， d， *J*=8.6 Hz， H-6）， 6.21 （1H， d， *J*=9.5 Hz， H-3）， 6.16 （1H， d， *J=*7.2 Hz， H-3″）， 5.47 （1H， d， *J*=4.8 Hz， H-3′）， 5.22 （1H， d， *J=*4.8 Hz， H-4′）， 2.00 （3H， d， *J*=7.2 Hz， H-4″）， 1.94 （3H， s， H-5″）， 1.51 （3H， s， 5′-CH_3_）， 1.44 （3H， s， 6′-CH_3_）。^13^C-NMR （CDCl_3_， 150 MHz）： *δ*_C_ 160.6 （C-2）， 112.4 （C-3）， 143.9 （C-4）， 128.7 （C-5）， 114.4 （C-6）， 156.0 （C-7）， 110.8 （C-8）， 154.1 （C-9）， 112.4 （C-10）， 77.6 （C-2′）， 72.4 （C-3′）， 59.9 （C-4′）， 25.6 （C-5′）， 22.4 （C-6′）， 166.9 （C-1″）， 139.3 （C-2″）， 127.2 （C-3″）， 20.5 （C-4″）， 15.8 （C-5″）。以上数据与文献报道基本一致^［15］^，将化合物3a和3b分别鉴定为（3′*S*，4′*S*）-3′-当归酰氧基-4′-羟基-3′，4′-二氢邪蒿内酯和（3′*R*，4′*R*）-3′-当归酰氧基-4′-羟基-3′，4′-二氢邪蒿内酯。

化合物4（4a/4b） 白色粉末；ESI-MS *m*/*z* 367.117 1 ［M+Na］^+^，分子式C_19_H_20_O_6_（理论值为*m*/*z* 367.115 2，误差+5.1×10^‒6^）。^1^H-NMR （CDCl_3_， 400 MHz）： *δ*_H_ 7.60 （1H， d， *J*=9.5 Hz， H-4）， 7.35 （1H， d， *J*=8.6 Hz， H-5）， 6.80 （1H， d， *J*=8.6 Hz， H-6）， 6.49 （1H， d， *J*=4.7 Hz， H-4′）， 6.22 （1H， d， *J=*9.5 Hz， H-3）， 6.10 （1H， m， *J*=7.2 Hz， H-3‴）， 4.08 （1H， d， *J=*4.7 Hz， H-3′）， 2.00 （3H， d， *J*=7.2 Hz， H-4‴）， 1.89 （3H， s， H-5‴）， 1.49 （3H， s， 5′-CH_3_）， 1.44 （3H， s， 6′-CH_3_）。^13^C-NMR （CDCl_3_， 100 MHz）： *δ*_C_ 160.0 （C-2）， 112.8 （C-3）， 143.3 （C-4）， 129.1 （C-5）， 114.4 （C-6）， 156.9 （C-7）， 107.2 （C-8）， 154.1 （C-9）， 112.1 （C-10）， 78.5 （C-2′）， 71.4 （C-3′）， 63.3 （C-4′）， 25.6 （C-5′）， 20.8 （C-6′）， 168.1 （C-1‴）， 138.8 （C-2‴）， 127.3 （C-3‴）， 20.2 （C-4‴）， 15.7 （C-5‴）。以上数据与文献报道基本一致^［15］^，将化合物4a和4b分别鉴定为（3′*S*，4′*S*）-3′-羟基-4′-当归酰氧基-3′，4′-二氢邪蒿内酯和（3′*R*，4′*R*）-3′-羟基-4′-当归酰氧基-3′，4′-二氢邪蒿内酯。

化合物5 白色粉末；ESI-MS *m*/*z* 425.119 2 ［M+Na］^+^，分子式为C_21_H_22_O_8_（理论值为*m*/*z* 425.120 7，误差‒3.5×10^‒6^）。^1^H-NMR （CDCl_3_， 400 MHz）： *δ*_H_ 7.60 （1H， d， *J=*9.5 Hz， H-4）， 7.36 （1H， d， *J*=8.6 Hz， H-5）， 6.81 （1H， d， *J*=8.6 Hz， H-6）， 6.61 （1H， d， *J*=4.8 Hz， H-4′）， 6.25 （1H， d， *J=*9.5 Hz， H-3）， 5.42 （1H， d， *J*=4.8 Hz， H-3′）， 3.05 （1H， q， *J*=5.4 Hz， H-3″）， 2.15 （3H， s， H-2‴）， 1.48 （3H， s， H-5″）， 1.45 （3H， s， 5′-CH_3_）， 1.41 （3H， s， 6′-CH_3_）， 1.32 （3H， d， *J=*5.4 Hz， H-4″）。^13^C-NMR （CDCl_3_， 100 MHz）： *δ*_C_ 170.1 （C-1″）， 169.1 （C-1‴）， 160.0 （C-2）， 156.7 （C-7）， 154.0 （C-9）， 143.5 （C-4）， 129.4 （C-5）， 114.6 （C-6）， 113.4 （C-3）， 112.7 （C-10）， 106.9 （C-8）， 77.6 （C-2′）， 71.4 （C-3′）， 60.9 （C-2″）， 60.7 （C-3″）， 59.7 （C-4′）， 25.3 （C-5′）， 22.9 （C-6′）， 20.8 （C-2‴）， 19.4 （C-5″）， 13.9 （C-4″）。以上数据与文献报道一致^［12］^，将化合物5鉴定为（3′*S*，4′*S*）-isoepoxypteryxin。

## 3 结论

本研究构建并验证了一种以“体外代谢产物导向-原药材靶向分离-代谢产物确证”的策略，成功解决了前胡中活性成分角型吡喃香豆素代谢研究中缺乏对照品的难题。利用该策略，本研究通过从前胡原药材中定向捕获并制备了9个对照品，促进前胡中APs的高效分离，其中，化合物1b、2b为已知平面结构的新构型。实现了对微量代谢产物（特别是立体异构体）的精准结构鉴定，阐明了（+）-PA与（+）-Pte在HLMs及体内的代谢路径。研究结果明确了水解、氧化及酰基迁移为APs的主要代谢途径，并揭示了（+）-PA与（+）-Pte的代谢差异。此方法不仅深化了APs的代谢机理，也为中药复杂成分微量代谢产物的结构鉴定与对照品获取提供了参考。
